# Clinical Determinants and Prognostic Implications of Right Ventricular Dysfunction in Pulmonary Hypertension Caused by Chronic Lung Disease

**DOI:** 10.1161/JAHA.118.011464

**Published:** 2019-01-16

**Authors:** Kurt W. Prins, Lauren Rose, Stephen L. Archer, Marc Pritzker, E. Kenneth Weir, Matthew D. Olson, Thenappan Thenappan

**Affiliations:** ^1^ Cardiovascular Division University of Minnesota Medical School Minneapolis MN; ^2^ Department of Medicine Queen's University Kingston Ontario Canada

**Keywords:** pulmonary hypertension, right ventricle, right ventricle echocardiography, right ventricular dysfunction, sex‐specific, Heart Failure, Pulmonary Hypertension

## Abstract

**Background:**

Patients with pulmonary hypertension caused by chronic lung disease (Group 3 PH) have disproportionate right ventricle (RV) dysfunction, but the correlates and clinical implications of RV dysfunction in Group 3 PH are not well defined.

**Methods and Results:**

We performed a cohort study of 147 Group 3 PH patients evaluated at the University of Minnesota. RV systolic function was quantified using right ventricular fractional area change (RVFAC) and ^+^ dP/dt_max_/instantaneous pressure. Tau and RV diastolic stiffness characterized RV diastolic function. Multivariate linear regression was used to define correlates of RVFAC. Kaplan‐Meier and Cox proportional hazards analyses were used to examine freedom from heart failure hospitalization and death. Positive correlates of RVFAC on univariate analysis were pulmonary arterial compliance, cardiac index, and left ventricular diastolic dimension. Conversely, male sex, N‐terminal pro‐brain natriuretic peptide, heart rate, right atrial enlargement, mean pulmonary arterial pressure, and pulmonary vascular resistance were negative correlates. Male sex was the strongest predictor of lower RVFAC, after adjusting for pulmonary vascular resistance and pulmonary arterial compliance. When comparing sexes, males had lower RVFAC (26% versus 31%, *P*=0.03) both overall and for any given mean pulmonary arterial pressure and pulmonary vascular resistance value. Males exhibited a reduction in ^+^ dP/dt_max_/instantaneous pressure as pulmonary vascular resistance increased, whereas females did not. There were no sex differences in RV diastolic function. RV dysfunction (RVFAC <28%) was associated with increased risk of heart failure hospitalization or death (hazard ratio: 1.84, 95% CI: 1.04–3.10, *P*=0.035).

**Conclusions:**

Male sex is associated with RV dysfunction in Group 3 PH, even after adjusting for RV afterload. RV dysfunction (RVFAC <28%) identifies Group 3 PH patients at risk for poor outcomes.

**Clinical Perspective**

**What Is New?**

- In our pulmonary hypertension caused by chronic lung disease cohort, male sex is independently associated with right ventricular dysfunction even after correcting for pulmonary vascular disease severity.
- Right ventricular dysfunction is associated with increased risk of heart failure hospitalization or death, highlighting its clinical significance in pulmonary hypertension caused by chronic lung disease.

**What Are the Clinical Implications?**

- Presence of right ventricular dysfunction identifies a high‐risk phenotype in pulmonary hypertension caused by chronic lung disease, and future therapies that target right ventricular dysfunction may improve outcomes in this vulnerable patient population.

## Introduction

Pulmonary hypertension caused by chronic lung disease (Group 3 PH) is the second leading cause of PH.^1^, ^2^ Group 3 PH patients have the worst long‐term survival of all the PH groups.^2^, ^3^ Moreover, Group 3 PH patients have higher medical costs^4^ and increased mortality when compared with patients with chronic lung disease without pulmonary hypertension.^5^, ^6^ Not only is Group 3 PH a costly and deadly condition, but the incidence and prevalence are increasing over time.^2^ Unfortunately, there are no effective medical therapies for Group 3 PH because traditional pulmonary arterial hypertension (PAH)–specific therapies do not augment exercise capacity or mitigate symptoms.^7^ Thus, Group 3 PH is a serious, difficult to treat, and growing public health problem. Hence, there is an unmet need to better understand this patient population to improve outcomes.

Recently, we showed that Group 3 PH patients have worse right ventricular (RV) systolic function than PAH patients despite having less severe pulmonary vascular disease, as determined by hemodynamic measures.^8^ The cause of the disproportionate RV systolic dysfunction in Group 3 PH is unknown. Better understanding of this observation may provide insight into pathological mechanisms, which could be used to devise new interventions to improve RV function. Moreover, the clinical consequences of RV dysfunction in Group 3 PH are not well described. Therefore, it is important to define the impact of impaired RV function in Group 3 PH because this may represent a novel therapeutic target for this vulnerable patient population.

Accordingly, we aimed to (1) describe the clinical correlates of RV dysfunction in Group 3 PH to identify variables that may provide insight to better understand the risk factors for disproportionate RV dysfunction in Group 3 PH, and (2) examine the impact of RV dysfunction on clinical outcomes in Group 3 PH.

## Methods

The data that support the findings of this study are available from the corresponding author upon reasonable request.

### Study Population

We studied adult patients (≥18 years of age) with Group 3 PH in the Minnesota Pulmonary Hypertension Repository.^9^ Briefly, the Minnesota Pulmonary Hypertension Repository is a prospective registry that collects specific variables on all consecutive patients treated at the University of Minnesota Pulmonary Hypertension Clinic (March 2014–present). Data are collected by chart review and entered using an internet‐based electronic data‐capture system. Patients diagnosed before March 2014 are entered retrospectively and those who are diagnosed on or after March 2014 are entered into the registry prospectively. All patients gave informed consent for repository participation. The Minnesota Pulmonary Hypertension Repository is approved by the University of Minnesota Institutional Review Board.

Group 3 PH was defined as a mean pulmonary arterial pressure (mPAP) ≥25 mm Hg at rest with a pulmonary capillary wedge pressure of ≤15 mm Hg in patients with 1 or more of the following lung conditions in the absence of other causes to explain PH:

1. Chronic obstructive lung disease: diagnosed by reduced expiratory flow rates (forced expiratory volume in 1‐s/forced vital capacity <70% predicted) and/or moderate‐to‐severe emphysema on a computerized tomography scan of the chest based on the visual classification system from the Fleischner Society.^10^
2. Interstitial lung disease: diagnosed by reduced total lung capacity <60% and/or evidence of moderate‐to‐severe interstitial fibrosis of lung parenchyma by computerized tomography scan of the chest.
3. Combined pulmonary fibrosis and emphysema: based on computerized tomography scan of the chest and pulmonary function test.
4. Obesity‐related lung disease: including obstructive sleep apnea (diagnosed by overnight sleep study) and obesity hypoventilation syndrome. Obesity was defined as body mass index >30 kg/m^2^.

We excluded patients with other World Health Organization categories of PH based on clinical evaluation and objective tests. Patients were excluded if they had idiopathic PAH, heritable PAH, drug‐induced PAH, or PAH associated with connective disease, liver disease, congenital heart disease, or HIV infection. Chronic pulmonary thromboembolic disease was excluded based on the findings on ventilation perfusion (V/Q) scan (high or intermediate probability), contrast‐enhanced chest computerized tomography, or pulmonary angiography if necessary. Patients with pulmonary venous hypertension, defined as mPAP ≥25 mm Hg at rest with a pulmonary capillary wedge pressure of >15 mm Hg, were also excluded.

### Clinical Characteristics

The following baseline variables at the time of referral were analyzed: age, sex, comorbid conditions, World Health Organization functional class, and medication use including PAH‐specific medications (prostacyclins, endothelin antagonists, phosphodiesterase inhibitors, and guanylate cyclase stimulators). Baseline laboratory tests included serum hemoglobin, serum creatinine, and serum N‐terminal pro‐brain natriuretic peptide. Pulmonary function testing and assessment of exercise capacity by 6‐minute walk test were also recorded. The Charlson Comorbidity Index^11^ was calculated to quantify burden of medical complexity.

### Echocardiographic Data

Echocardiography was performed using a commercially available Philips system (iE33, Philips Ultrasound, Bothell, WA) with a 3.5‐MHz multiphase‐array probe. The baseline echocardiographic variables collected on all available patients included the following: left ventricular ejection fraction, dimensions and mass, presence of left or right atrial (RA) enlargement, RV size and function, and presence of pericardial effusion. RV size was semiquantitatively described as normal size (two thirds or less of the left ventricular size) or as mildly (RV similar size as the left ventricle size), moderately (RV larger than the left ventricle), or severely enlarged (RV much larger than the left ventricle), using visual estimations as previously described.^12^ RV size was quantified using end‐diastolic and end‐systolic areas measured from the apical 4‐chamber view.

### Hemodynamic Data

Patients underwent right heart catheterization, as clinically indicated by echocardiogram showing pulmonary hypertension or RV dysfunction or worsening dyspnea, in the University of Minnesota cardiac catheterization laboratory. Hemodynamics were obtained using a 7‐Fr, balloon‐tipped, flow‐directed catheter placed either into the internal jugular vein or the common femoral vein. The following hemodynamic variables were recorded at the end of expiration: RA pressure, RV systolic and end‐diastolic pressures, systolic pulmonary artery pressure, diastolic pulmonary artery pressure, mPAP, and pulmonary capillary wedge pressure. Cardiac output was determined as the mean of 3 measurements with the thermodilution method or indirect Fick method based on total body oxygen consumption, as estimated via the formula of LaFarge and Miettinen.^13^ Pulmonary vascular resistance (PVR) was calculated in Wood units as the difference between mPAP and pulmonary capillary wedge pressure divided by the cardiac output. Pulmonary arterial compliance (PAC) (mL/mm Hg) was calculated as the ratio of stroke volume to the pulmonary artery pulse pressure, as previously described.^14^ Acute vasodilator response was assessed during right heart catheterization with 80 PPM of inhaled nitric oxide for 5 minutes. A positive vasodilator response was defined as a >10 mm Hg reduction of mPAP to a mPAP of <40 mm Hg, with an unchanged or improved cardiac index.^15^

### RV Function Assessment

We quantified RV function by calculating right ventricular fractional area change (RVFAC), S’ velocity, and tricuspid annular plane systolic excursion (TAPSE) using echocardiography.^16^ RVFAC was measured by manually tracing the RV endocardium in the apical 4‐chamber view in systole and diastole, as per the American Society of Echocardiography guidelines.^16^ Measurements were performed in all patients by a single, blinded investigator (K.W.P.) who had access to all raw echocardiographic images. K.W.P. performed over‐reads of all echocardiograms because RVFAC is not routinely reported in our practice. Intraobserver variability was calculated by repeated RVFAC measurement in all patients by K.W.P. If patients did not have M‐mode measurement of TAPSE recorded, we measured TAPSE by postprocessing of 2‐dimensional 4‐chamber images using a Java‐based imaging software program (Image J, National Institutes of Health, Bethesda, MD), as described previously.^17^ Complete echocardiographic data were not available for some study patients because of unsatisfactory image quality or because the echocardiogram was performed at the referring center and was unavailable for analysis (n=58 unavailable for RVFAC analysis). For a subset of patients who had available raw data from RV pressure tracings collected during right heart catheterization (those diagnosed from 2014 onwards), ^+^dp/dt_max_, ^−^dp/dt_min_, and the instantaneous pressure (IP) at ^+^dp/dt_max_ were calculated using a custom‐made program on LabView (Austin, TX). Values for each variable were calculated as the average of 3 cardiac cycles. RV contractility was estimated using ^+^dp/dt_max_/IP to adjust for differences in preload.^18^

To quantify RV diastolic function, we calculated Tau in the subset of patients who had RV pressure tracings from right heart catheterization using a custom‐made program in LabView, based on the formula described by Weiss et al.^19^ In addition, RV diastolic stiffness was calculated as the ratio of change in RV pressure during diastole obtained from right heart catheterization over change in RV area determined with echocardiography, as previously described.^20^

### Vital Statistics

All patients were followed at the University of Minnesota PH clinic every 3 to 6 months. Vital statistics were obtained for all patients by chart review and Minnesota Death index. For each death, the date and cause of death was collected. In all patients who were not identified as deceased using the Minnesota Death Index, it was possible to establish vital status by chart review. Heart failure hospitalizations were collected for all patients and independently confirmed by chart review of the encounter by L.R.

### Statistical Analysis

Categorical data were expressed as frequency and proportions, whereas continuous data were presented as mean±SD unless otherwise indicated. Unpaired *t* tests or Wilcoxon–Mann–Whitney test were used to compare means of 2 groups with continuous variables as appropriate. Chi square or Fisher exact test were performed to compare proportions for categorical variables as appropriate. We used RVFAC to define reduced RV function, to determine the clinical correlates, and to compare clinical outcomes because it is more strongly associated with RV ejection fraction (RVEF) measured using cardiac magnetic resonance imaging (MRI) than TAPSE, especially in patients with severe pulmonary vascular disease.^21^ To understand the determinants of RV function, we performed univariate and multivariable linear regression analyses with RVFAC as the dependent variable. In the multivariable model, RV afterload was adjusted for PVR and PAC. Survival analysis was performed using the Kaplan–Meier method, with entry into the study defined as the date of diagnostic right heart catheterization. The primary end point was a combined event of either heart failure hospitalization or all‐cause mortality. Patients were censored at lung transplantation or study completion (June 1, 2018). Patients were categorized into 2 groups: normal and reduced RV function groups based on the median RVFAC. The survival between groups was compared using log‐rank test. Cox's proportional hazards analyses were performed to determine the hazard ratio (HR) for death or heart failure hospitalization with reduced RV function. Because lung transplantation represents a competing risk, to ensure robustness, we repeated the analysis by considering lung transplant as a competing risk using the Fine Gray method.^22^, ^23^ Sensitivity analysis was performed by excluding patients who underwent lung transplantation and by considering lung transplant as a primary event. Comparison of best‐fit lines was performed to determine the differences in response to RV function as load increased using GraphPad Version 7 (La Jolla, CA).

All statistical analyses were performed using Stata software Version 10 and 15 (Stata Corp LP, College Station, TX) or GraphPad Version 7. A *P* value of <0.05 was considered statistically significant.

## Results

We studied 147 consecutive Group 3 PH patients at the University of Minnesota Pulmonary Hypertension Clinic. The clinical, echocardiographic, and hemodynamic characteristics of the cohort are described in Table 1. Briefly, the mean age of the study cohort was 65±11 years and 48% of the patients were male. Ninety percent of patients had significant functional impairment, defined as World Health Organization Functional class III or IV. PH was associated with chronic obstructive lung disease in 57 patients (39%), interstitial lung disease in 64 patients (43%), obesity‐related in 13 patients (9%), and combined pulmonary fibrosis and emphysema in 13 patients (9%). There were multiple comorbid conditions with the most prevalent being systemic hypertension. Twenty patients (14%) had connective tissue disease. In these patients, the treating clinician made the determination that parenchymal lung disease, related to the underlying connective tissue disease, was the main driver of PH. The mean age‐adjusted Charlson Comorbidity Index was 5.0±2.3. The cohort had significant pulmonary vascular disease as demonstrated by the mPAP of 39±10 mm Hg, mean PVR of 5.9±2.9 Wood units, and mean PAC of 2.0±1.1 mL/mm Hg (Table 1). The results of pulmonary function tests for this cohort have been described previously.^24^

**Table 1:** Clinical, Echocardiographic, and Hemodynamic Characteristics of Patients With Pulmonary Hypertension Caused by Chronic Lung Disease

Characteristics	n=147
Age, y	65±11
Male, n (%)	71 (48)
Body mass index, kg/m^2^	30±8
WHO functional class (n=122), n (%)	
II	12 (10)
III	95 (78)
IV	15 (12)
Comorbidities, n (%)	
Hypertension	104 (71)
Diabetes mellitus	41 (28)
Hyperlipidemia	77 (52)
Coronary artery disease	44 (30)
Atrial fibrillation	30 (20)
Connective tissue disease	20 (14)
Charlson Comorbidity Index	5.0±2.3
Cause of lung disease	
Chronic obstructive pulmonary disease	57 (39)
Interstitial lung disease	64 (43)
Obesity‐related lung disease	13 (9)
Combined pulmonary fibrosis and emphysema	13 (9)
Medications, n (%)	
Oxygen	92 (63)
Diuretics	73 (50)
Digoxin	12 (8)
Warfarin	20 (14)
Calcium channel blockers	30 (20)
Phosphodiesterase‐5 inhibitors	14 (10)
Endothelin receptor antagonists	2 (1)
Prostacyclin	0 (0)
Six‐min walk test	
Distance, m (n=87)	236±105
Rest oxygen saturation, % (n=85)	97±2
Peak exercise oxygen saturation, % (n=84)	88±5
Pulmonary function test, % predicted	
FEV1 (n=120)	55±23
FVC (n=119)	63±22
FEV1/FVC (n=119)	69±19
TLC (n=74)	81±25
DL_CO_ (n=96)	36±19
Laboratory	
Serum hemoglobin, g/dL (n=146)	13.5±2.1
Serum creatinine, mg/dL (n=145)	0.9 (0.7–1.1)
Serum NT‐proBNP, pg/dL (n=123)	996 (234–3270)
Echocardiography	
Left ventricular EF, % (n=140)	60±9
Left ventricular mass index, g/m^2^ (n=107)	155±55
Left ventricular end diastolic diameter, cm (n=124)	4.3±0.7
Left atrial diameter, cm (n=92)	4.0±0.9
Left atrial volume index, mL/m^2^ (n=94)	29±12
Right ventricular enlargement, n (%)	96 (70)
Right ventricular end‐diastolic area, cm^2^, (n=89)	32±10
Right ventricular end‐systolic area, cm^2^ (n=89)	23±9
Right atrial enlargement, n (%)	77 (57)
Pericardial effusion, n (%)	12 (9)
Hemodynamics (n=147)	
Heart rate, beats/min	79±15
Mean right atrial pressure, mm Hg	7±4
Mean pulmonary artery pressure, mm Hg	39±10
Pulmonary capillary wedge pressure, mm Hg	10±3
Cardiac output, L/min	4.8±1.5
Cardiac index, L/min per m^2^	2.5±0.8
Pulmonary vascular resistance, Wood units	5.9±2.9
Diastolic pulmonary gradient, mm Hg	15±8
Pulmonary arterial compliance, mL/mm Hg	2.0±1.1
Vasodilator response, %	5 (3.4)
RV function	
RV fractional area change, % (n=89)	29±10
TAPSE, cm (n=99)	1.8±0.4
S’, cm/s (n=73)	10.9±2.7
^+^dp/dt_max_, mm Hg/s (n=60)	475±163
^+^dp/dt_max_/IP, s^−1^ (n=60)	16.0±6
^−^dp/dt_min_, mm Hg/s (n=60)	−521.0±147
Tau, ms (n=60)	49.1±18.0
Right ventricular diastolic stiffness, mm Hg/cm^2^ (n=68)	1.1±0.7

DLCO indicates diffusion capacity of the lung for carbon monoxide; EF, ejection fraction; FEV1, forced expiratory volume in 1 s; FVC, forced vital capacity; IP, instantaneous pressure; NT‐proBNP, N‐terminal prohormone of brain natriuretic peptide; RV, right ventricular; TAPSE, tricuspid plane annular systolic excursion; TLC, total lung capacity; WHO, World Health Organization.

### RV Function

**Figure 1:**
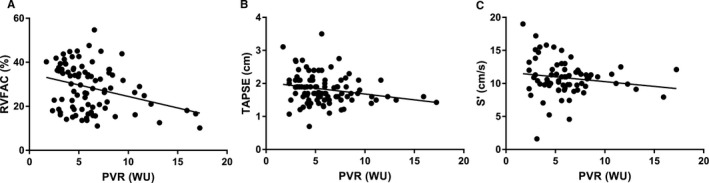
Right ventricular fractional area change (RVFAC) is more strongly associated with pulmonary vascular resistance (PVR) than tricuspid annular plane systolic excursion (TAPSE) or S’. **A**, There is a significant inverse relation between RVFAC and PVR (*r*=−0.31, *P*=0.003). **B**, TAPSE is inversely associated with PVR (*r*=−0.22, *P*=0.027). **C**, S’ and PVR are not significantly related (*r*=−0.16, *P*=0.18). WU indicates Wood units.

RV systolic function was mild‐to‐moderately reduced with a mean RVFAC 29±10% (normal >35%^16^; n=89), TAPSE (1.8±0.4 cm [normal >1.7 cm^16^]; n=99), and S’ 10.9±2.7 cm/s (normal >9.5 cm/s^16^; n=73). Of these echocardiographic measures, RVFAC had the strongest relationship with PVR (Figure 1). This is congruent with cardiac MRI analysis showing RVFAC is more strongly associated with RVEF than TAPSE, especially in patients with severe pulmonary vascular disease.^21^ The intraobserver variability in the measurement of RVFAC was low with a correlation coefficient of 0.95 and a mean difference of −0.6±3.3%. Thus, we used RVFAC as the most sensitive measure of RV systolic function to assess the clinical determinants and prognostic importance of RV function. The mean ^+^dp/dt_max_/IP was 16.0±6.2/s. RV diastolic function was impaired with a mean Tau of 49.1±18.0 ms (normal 31±13^25^) and RV diastolic stiffness of 1.1±0.7 mm Hg/cm^2^ (normal 0.7±0.3 mm Hg/cm^2^ 20).

### Clinical Correlates of RV Dysfunction

To define the correlates of RV dysfunction, we performed univariate and multivariate linear regression analysis using RVFAC as the dependent variable. On univariate analysis, as expected, RVFAC was associated with parameters of RV afterload. Lower PAC, higher mPAP, and higher PVR were associated with lower RVFAC. In addition, on univariate analysis, cardiac index and left ventricular internal diameter in diastole were associated with higher RVFAC. Elevated heart rate, serum N‐terminal pro‐brain natriuretic peptide, serum hemoglobin, male sex, higher forced expiratory volume in 1 s, and the presence of RA and RV enlargement were associated with lower RVFAC (Table 2). In a multivariate model adjusted for RV afterload (PVR and PAC), male sex, higher serum N‐terminal pro‐brain natriuretic peptide, RA and RV enlargement, and higher heart rate were associated with lower RVFAC (Table 2). Male sex was the strongest predictor of RVFAC after adjusting for RV afterload (Table 2).

**Table 2:** Clinical Determinants of RV Fractional Area Change

Characteristics	Univariate Predictors β‐Coefficient (95% CI)	*P* Value	Adjusted for RV Afterload (PVR & PAC) β‐Coefficient (95% CI)	*P* Value
Male	−5.2 (−0.9 to −9.4)	0.018	9.0 (4.4–13.6)	<0.001
Serum hemoglobin	−0.1 (−1.9 to −0.02)	0.045	−0.8 (−1.8 to 0.2)	0.108
Log NT‐proBNP	−3.3 (−4.6 to −2.0)	<0.001	−2.8 (−4.4 to −1.2)	0.001
FEV1	−0.1 (−0.2 to −0.01)	0.027	−0.1 (−0.2 to 0.03)	0.128
RA enlargement	−7.2 (−11.7 to −2.7)	0.002	−5.4 (−10.7 to −0.1)	0.046
RV enlargement	−7.2 (−12.1 to −2.3)	0.005	−6.7 (−12.0 to −1.3)	0.016
LVEDD	3.3 (0.1–6.4)	0.044	1.2 (−2.6 to 5.1)	0.526
Heart rate	−0.3 (−0.4 to −0.1)	0.002	−0.2 (−0.4 to −0.05)	0.012
Cardiac index	2.9 (0.2–5.6)	0.037	1.1 (−2.4 to 4.7)	0.525
mPAP	−0.4 (−0.6 to −0.2)	<0.001		
PVR	−1.0 (−1.7 to −0.4)	0.003		
PAC	3.4 (1.1–5.6)	0.004		

Multivariate predictors are adjusted for pulmonary vascular resistance (PVR) and pulmonary arterial compliance (PAC). FEV1 indicates forced expiratory volume in 1 s; FVC, forced vital capacity; NT‐proBNP, N‐terminal prohormone of brain natriuretic peptide; LVEDD, left ventricular end diastolic diameter; RA, right atrial; RV, right ventricular.

### Comparing Males and Females

To further examine how sex affected RV function, we first conducted a comprehensive clinical comparison of males and females. Overall, males were slightly older and had more comorbid conditions including atrial fibrillation and coronary artery disease, but there was no significant difference in Charlson Comorbidity scores (Table 3). There were no differences in World Health Organization functional class, 6‐minute walk distance, or pulmonary function tests, but males had lower nadir oxygen saturation during the 6‐minute walk test (Table 3). On laboratory examination, males had higher creatinine and hemoglobin. Males also had higher left ventricular mass and higher RV end systolic and end diastolic areas as determined by echocardiography (Table 3).

**Table 3:** Comparison of Clinical Characteristics of Patients With Pulmonary Hypertension Caused by Chronic Lung Disease Based on Sex

Characteristics	Male (n=71)	Female (n=76)	*P* Value
Age, y	67±10	63±12	0.068
Body mass index, kg/m^2^	31±8	30±7	0.529
WHO functional class (n=122), n (%)			0.365
II	3 (5)	9 (13)	
III	45 (82)	50 (75)	
IV	7 (13)	8 (12)	
Comorbidities, n (%)			
Hypertension	53 (75)	51 (67)	0.315
Diabetes mellitus	23 (32)	18 (24)	0.239
Hyperlipidemia	41 (58)	36 (47)	0.208
Coronary artery disease	28 (39)	16 (21)	0.015
Atrial fibrillation	22 (31)	8 (11)	0.002
Charlson Comorbidity Index	5.3±2.4	4.8±2.2	0.186
Medications, n (%)			
Oxygen	47 (66)	45 (59)	0.382
Diuretics	38 (54)	35 (46)	0.365
Digoxin	9 (13)	3 (4)	0.071
Coumadin	13 (19)	7 (9)	0.108
Calcium channel blockers	13 (18)	17 (22)	0.542
Phosphodiesterase‐5 inhibitors	6 (9)	8 (11)	0.782
Endothelin receptor antagonists	1 (1)	1 (1)	1.000
Prostacyclins	0 (0)	0 (0)	···
Six‐min walk test			
Distance, m (n=87)	239±113	233±100	0.793
Rest oxygen saturation, % (n=85)	97±2	98±2	0.067
Nadir exercise oxygen saturation, % (n=84)	86±5	88±5	0.043
Pulmonary function test, % predicted			
FEV1 (n=120)	55±25	54±22	0.830
FVC (n=119)	64±22	62±22	0.588
FEV1/FVC (n=119)	67±19	70±20	0.412
TLC (n=74)	79±27	83±24	0.492
DL_CO_ (n=96)	35±16	37±21	0.555
Laboratory			
Serum hemoglobin, g/dL (n=146)	14.1±2.2	13.0±1.9	0.002
Serum creatinine, mg/dL (n=145)	1.0 (0.8–1.3)	0.8 (0.6–0.9)	<0.001
Serum NT‐proBNP, pg/dL (n=123)	1442 (202–3304)	751 (245–3108)	0.602
Echocardiography			
Left ventricular EF, % (n=140)	59±10	62±8	0.087
Left ventricular mass index, g/m^2^ (n=107)	170±58	141±47	0.007
Left ventricular end‐diastolic diameter, cm (n=124)	4.4±0.7	4.2±0.7	0.093
Left atrial diameter, mm (n=92)	4.1±0.9	3.9±0.8	0.144
Left atrial volume index, mL/m^2^ (n=94)	32±13	27±9	0.062
RV enlargement (n=137), n (%)	53 (78)	42 (62)	0.046
RV end‐diastolic area, cm^2^ (n=89)	36±8	28±10	<0.001
RV end‐systolic area, cm^2^ (n=89)	27±7	20±9	<0.001
Right atrial enlargement (n=136), n (%)	43 (64)	34 (49)	0.080
Pericardial effusion (n=138), n (%)	6 (9)	6 (8)	1.000
Hemodynamics			
Heart rate, beats/min (n=121)	78±16	79±15	0.956
Mean right atrial pressure, mm Hg (n=145)	8±5	7±4	0.184
Mean pulmonary artery pressure, mm Hg (n=147)	40±10	38±10	0.403
Pulmonary capillary wedge pressure, mm Hg (n=143)	10±3	10±3	0.140
Cardiac output, L/min (n=145)	5.0±1.6	4.5±1.4	0.043
Cardiac index, L/min per m^2^ (n=141)	2.5±0.9	2.5±0.8	0.873
Pulmonary vascular resistance, WU (n=147)	4.7±1.5	7.0±3.4	<0.001
Diastolic pulmonary gradient, mm Hg (n=143)	16±8	14±8	0.202
Pulmonary arterial compliance, mL/mm Hg (n=120)	2.2±1.2	1.8±1.0	0.084
Vasodilator response, % (n=88)	4 (6)	1 (1)	0.197
RV function			
RV fractional area change (n=89)	26±9	31±11	0.028
TAPSE, cm (n=99)	1.8±0.5	1.8±0.4	0.934
S’, cm/s (n=73)	10.6±3.2	11.1±2.0	0.413
^+^dp/dt_max_, mm Hg/s (n=60)	452±132	494±182	0.327
^+^dp/dt_max_/IP, s^−1^ (n=60)	16.6±7.2	15.6±5.2	0.537
^−^dp/dt_min_, mm Hg/s (n=60)	−492±145	−545±145	0.164
Tau, ms (n=60)	49.6±18.9	48.7±17.4	0.856
RV diastolic stiffness, mm Hg/cm^2^ (n=68)	1.1±0.7	1.1±0.6	0.816

DL_CO_ indicates diffusion capacity of the lung for carbon monoxide; EF, ejection fraction; FEV1, forced expiratory volume in 1 s; FVC, forced vital capacity; NT‐proBNP, N‐terminal prohormone of brain natriuretic peptide; RV, right ventricular; TAPSE, tricuspid plane annular systolic excursion; TLC, total lung capacity; WHO, World Health Organization; WU, Wood units.

When comparing invasive hemodynamics, females had a significantly higher PVR (Table 3) and a trend for lower PAC (Table 3), suggesting there was more severe pulmonary vascular disease in females. Males had a higher cardiac output, but there was no difference in cardiac index between sexes (Table 3). Finally, there was no difference in proportion of vasodilator‐responsive patients between sexes (Table 3).

### Males Have Worse RV Systolic Function Than Females

**Figure 2:**
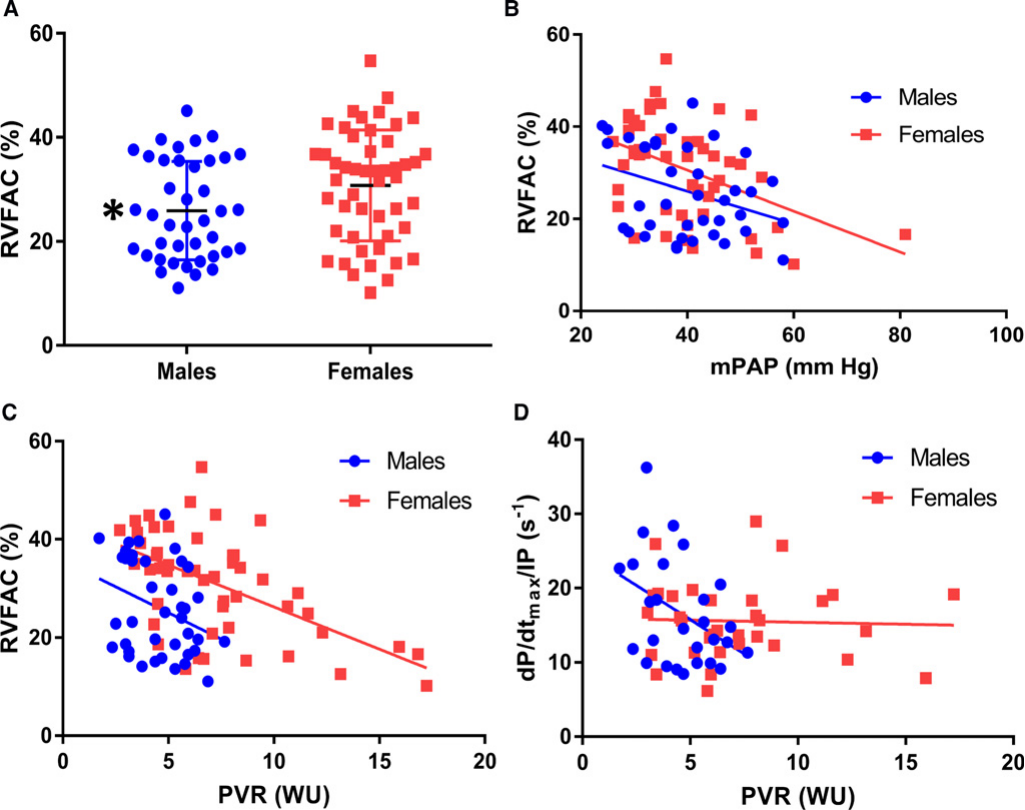
In Group 3 pulmonary hypertension, males have worse right ventricular (RV) systolic function than females. **A**, Males have a significantly lower right ventricular fractional area change (RVFAC) than females (26±9% vs 31±11, *P*=0.02). **B**, When RVFAC was plotted vs mean pulmonary artery pressure (mPAP), males had lower RVFAC for every mPAP as demonstrated by a significant different y‐intercept (males: 40.1±40.8, females: 48.3±37.9 *P*=0.02). **C**, When the relationship between RVFAC and pulmonary vascular resistance (PVR) was examined, males had a lower RVFAC at all PVRs as evidenced by a significantly different y‐intercept (males: 35.6±30.4, females: 43.4±21.1 *P*<0.0001). **D**, In males, ^+^dp/dt_max_/instantaneous pressure (IP) decreased as PVR increased (*r*=−0.41, *P*=0.04) while in females it did not change significantly (*r*=−0.04, *P*=0.84). The slope of the regression lines was significantly different when males and females were compared (males: −1.8±4.3, females: −0.05±1.5, *P*=0.03). WU indicates Wood units.

Overall, males had significantly lower RVFAC than females (26±9% versus 31±11, *P*=0.03) (Figure 2A). When RVFAC was plotted versus mPAP, males had a lower RVFAC for all mPAP (Figure 2B, *P*=0.02). Similarly, when RVFAC was plotted against PVR, males had a significantly lower RVFAC at all PVR (Figure 2C, *P*<0.0001). There was no sex difference in RV contractility (^+^dp/dt_max_/IP) between males and females (males: 16.6±7.2 [n=27] females: 15.6±5.2 [n=33], *P*=0.54). However, when ^+^dp/dt_max_/IP was plotted against PVR, females maintained ^+^dp/dt_max_/IP as PVR increased (*r*=−0.04, *P*=0.84), but in males, ^+^dp/dt_max_/IP dropped as PVR increased (*r*=−0.41, *P*=0.04) (Figure 2D). Furthermore, the slopes of the 2 best‐fit lines were significantly different (*P*=0.03), providing evidence of sex‐based divergent responses of RV contractility to increasing afterload.

### No Differences in RV Diastolic Indices Between Males and Females

**Figure 3:**
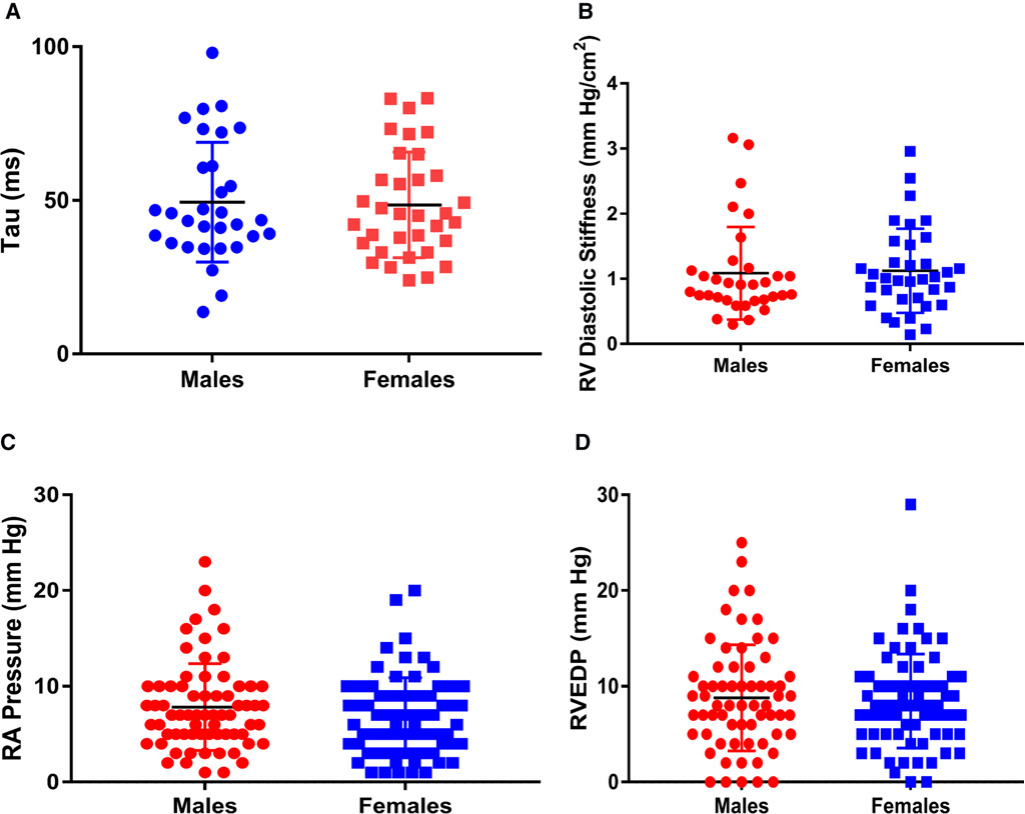
No differences in right ventricular (RV) diastolic function comparing males and females. Tau (males: 49.5±19.7 ms, females: 48.5±16.9 ms, *P*=0.82) (**A**), RV diastolic stiffness (males: 1.1±0.30 mm Hg/cm^2^, females: 1.1±0.65 mm Hg/cm^2^, *P*=0.82) (**B**), right atrial (RA) pressure (males: 7.8±4.5 mm Hg, females: 6.8±4.0 mm Hg, *P*=0.16) (**C**), and RV end‐diastolic pressure (RVEDP) (males: 8.8±5.5 mm Hg, females 8.5±4.9 mm Hg, *P*=0.70) (**D**) did not differ when males and females were compared.

Because there were sex differences in systolic function, we next compared measures of RV diastolic function. There were no significant differences in Tau (males: 49.5±19.7 ms, females: 48.5±16.9 ms, *P*=0.82), RV diastolic stiffness (males: 1.1±0.30 mm Hg/cm^2^, females: 1.1±0.65 mm Hg/cm^2^, *P*=0.82), RA pressure (males: 7.8±4.5 mm Hg, females: 6.8±4.0 mm Hg, *P*=0.16), and RV end‐diastolic pressure (males: 8.8±5.5 mm Hg, females 8.5±4.9, *P*=0.70) between males and females (Figure 3).

### RV Dysfunction Is Associated With Increased Risk of Heart Failure Hospitalization and Death

**Figure 4:**
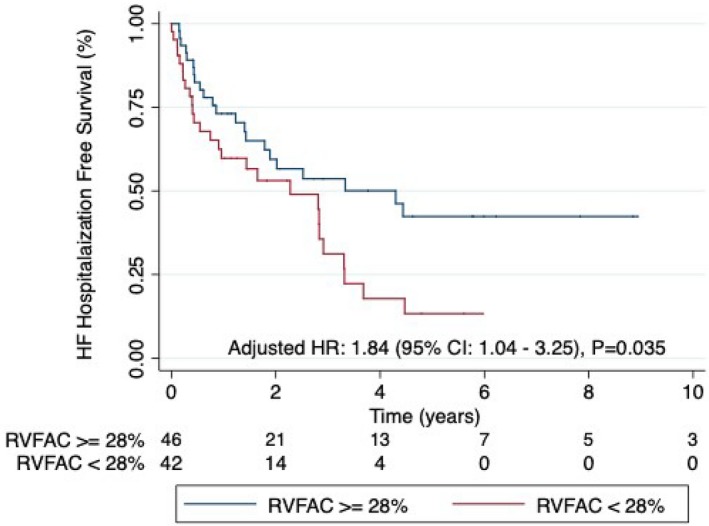
Patients with right ventricular (RV) dysfunction are at increased risk of the combined end point of hospitalization for heart failure (HF) or death. When the cohort was divided by the median RV fractional area change (RVFAC) of 28%, patients with RV dysfunction had more clinical events than those with preserved RV function. Hazard Ratio (HR) adjusted for Charlson Comorbidity Index: 1.84, 95% CI: 1.04–3.25, *P*=0.035.

We analyzed how RV dysfunction impacted clinical outcomes in Group 3 PH. When the cohort was divided by median RVFAC (28%), patients with RV dysfunction had similar functional impairments, 6‐minute walk distance, and medical treatment. However, patients with RV dysfunction had higher forced expiratory volume in 1 s on pulmonary function testing and higher serum hemoglobin and N‐terminal pro‐brain natriuretic peptide on laboratory investigation. On echocardiography examination, patients with impaired RV function had more RV and RA dilation and were more likely to have pericardial effusion. Patients with RV dysfunction had more severe pulmonary vascular disease as demonstrated by a higher mPAP and PVR and lower PAC (Table 4). Over a median follow‐up time of 1.4 years, 78 patients had a combined end point of either death or heart failure hospitalizations, 7 patients had lung transplant, and 1 patient was lost to follow‐up. When patients were categorized by the median RVFAC, patients with reduced RV function (RVFAC <28%) were at higher risk of a composite end point of death or heart failure hospitalizations when compared with patients with normal RV function (HR adjusted for Charlson Comorbidity Index: 1.84 [95% CI: 1.04–3.25], *P*=0.035) (Figure 4). In the Fine Gray competing risk analysis, the HR adjusted for Charlson's comorbidity index for a composite end point of death or heart failure hospitalizations in patients with reduced RV function was 1.79 (95% CI: 1.04–3.10), *P*=0.035. Sensitivity analysis performed after excluding patients who underwent lung transplant (HR adjusted for Charlson comorbidity index: 1.83 [95% CI: 1.04–3.23, *P*=0.036]) or by considering lung transplant as a primary end point (HR adjusted for Charlson Comorbidity Index: 1.81 [95% CI: 1.05–3.13, *P*=0.032]) yielded similar results.

**Table 4:** Comparison of Patients With Normal and Reduced RV Function

Characteristics	Normal RV Function (n=43)	RV Dysfunction (n=46)	*P* Value
Age, y	64±12	68±9	0.118
Male, n (%)	16 (35)	25 (58)	0.027
Body mass index, kg/m^2^	29±7	30±6	0.350
WHO functional class (n=74), n (%)			0.509
II	5 (13)	2 (6)	
III	28 (74)	30 (83)	
IV	5 (13)	4 (11)	
Comorbidities, n (%)			
Hypertension	36 (78)	29 (67)	0.250
Diabetes mellitus	10 (22)	16 (37)	0.109
Hyperlipidemia	21 (46)	26 (61)	0.162
Coronary artery disease	13 (28)	14 (33)	0.659
Atrial fibrillation	10 (22)	11 (26)	0.670
Charlson Comorbidity Index	5.3±2.5	5.2±2.4	0.924
Medications, n (%)			
Oxygen	27 (59)	28 (65)	0.533
Diuretics	22 (48)	25 (58)	0.330
Digoxin	3 (7)	4 (9)	0.708
Warfarin	5 (11)	7 (16)	0.455
Calcium channel blockers	11 (24)	7 (16)	0.370
Phosphodiesterase‐5 inhibitors	7 (15)	4 (9)	0.524
Endothelin receptor antagonists	0 (0)	2 (5)	0.231
Prostacyclins	0 (0)	0 (0)	···
Six‐min walk test			
Distance, m (n=52)	233±101	220±109	0.666
Rest oxygen saturation, % (n=51)	97±3	97±2	0.773
Nadir exercise oxygen saturation, % (n=51)	87±5	87±6	0.998
Pulmonary function test, % predicted			
FEV1 (n=74)	50±20	63±23	0.014
FVC (n=74)	60±22	70±21	0.051
FEV1/FVC (n=74)	67±21	71±16	0.395
TLC (n=47)	82±30	85±23	0.679
DL_CO_ (n=60)	34±14	37±23	0.650
Laboratory			
Serum hemoglobin, g/dL (n=89)	12.9±1.9	14.2±2.5	0.007
Serum creatinine, mg/dL (n=88)	0.9 (0.7–1.2)	0.9 (0.8–1.2)	0.997
Serum NT‐proBNP, pg/dL (n=74)	391 (166–2773)	2693 (1604–6050)	<0.001
Echocardiography			
Left ventricular EF, % (n=88)	62±8	59±10	0.077
Left ventricular end diastolic diameter, cm (n=70)	4.5±0.7	4.2±0.7	0.132
Left ventricular mass index, g/m^2^ (n=70)	153±53	154±57	0.924
Left atrial diameter, cm (n=57)	3.9±1.0	4.3±0.7	0.168
Left atrial volume index, mL/m^2^ (n=64)	28±11	29±14	0.612
RV enlargement (n=87), n (%)	28 (64)	38 (88)	0.007
RV end‐diastolic area, cm^2^ (n=89)	28±9	36±9	<0.001
RV end‐systolic area, cm^2^ (n=89)	18±6	29±8	<0.001
Right atrial enlargement (n=85), n (%)	23 (52)	34 (83)	0.003
RV FAC% (n=89)	37±5	19±5	<0.001
Pericardial effusion, % (n=86)	1 (3)	8 (18)	0.030
TAPSE, cm (n=71)	2.0±0.4	1.6±0.5	<0.001
S’, cm/s (n=50)	11.8±2.8	9.5±2.1	0.002
Hemodynamics			
Heart rate, beats/min (n=74)	77±13	84±14	0.019
Mean right atrial, mm Hg (n=87)	7±3	8±5	0.157
Mean pulmonary arterial pressure, mm Hg (n=89)	37±8	43±11	<0.001
Pulmonary capillary wedge pressure, mm Hg (n=88)	11±3	10±4	0.349
Cardiac output, L/min (n=89)	4.8±1.4	4.5±1.4	0.293
Cardiac index, L/min per m^2^ (n=87)	2.6±0.8	2.3±0.7	0.093
Pulmonary vascular resistance, WU (n=89)	5.3±2.1	6.9±3.7	0.016
Diastolic pulmonary gradient, mm Hg (n=88)	12±7	18±9	<0.001
Pulmonary arterial compliance, mL/mm Hg (n=74)	2.1±1.1	1.5±0.9	0.012
Vasodilator response, % (n=89)	1 (2)	1 (2)	0.736

DL_CO_ indicates diffusion capacity of the lung for carbon monoxide; EF, ejection fraction; FAC, fractional area change; FEV1, forced expiratory volume in 1 s; FVC, forced vital capacity; NT‐proBNP, N‐terminal prohormone of brain natriuretic peptide; RV, right ventricular; TAPSE, tricuspid plane annular systolic excursion; TLC, total lung capacity; WHO, World Health Organization; WU, Wood units.

## Discussion

In this study, we defined the correlates of RV function and the impact of RV dysfunction on clinical outcomes in a cohort of Group 3 PH patients. We show that male sex is the strongest predictor of RV systolic dysfunction even after adjusting for RV afterload in Group 3 PH patients. Male patients have lower RVFAC at all mPAP and PVR than female patients. When assessing the response of RV contractility to increasing PVR, males drop ^+^dp/dt_max_/IP, a surrogate marker of intrinsic RV contractility,^18^ as PVR increases, whereas females retain RV contractility. In contrast, regarding RV diastolic function data, there are no differences in Tau, RV diastolic stiffness, RA pressure, and RV end‐diastolic pressure between males and females. Finally, Group 3 PH patients with RV dysfunction are at a higher risk of a combined end point of heart failure hospitalization or death, demonstrating that RV dysfunction is associated with worse outcomes in Group 3 PH.

Our finding that males have worse RV function than females in patients with Group 3 PH is novel, in that this form of PH has not been studied, but is entirely consistent with prior reports of sex‐based differences in RV function in normal subjects and patients with PAH (Group 1 PAH) and heart failure with preserved ejection fraction (Group 2 PH). In the MESA (Multi‐Ethnic Study of Atherosclerosis) cohort, cardiac MRI examination of 4204 patients revealed that males have a significantly lower RVEF than females, in the absence of pulmonary hypertension.^26^ In a cohort study of 63 PAH patients, male sex is associated with a lower RVEF as determined by equilibrium radionucleotide angiography.^27^ In 101 PAH patients treated at the VU University Medical Centre, males do not improve RVEF, as quantified by cardiac MRI, as much as their female counterparts after treatment with PAH‐specific therapy, which partially explains the worse survival in men with PAH.^28^ In a study of 40 idiopathic PAH patients, males have lower cardiac MRI‐derived RVEF despite having similar mPAP and indexed PVR.^29^ In 96 heart failure with preserved ejection fraction patients evaluated at the Mayo Clinic, male sex is associated with lower RVFAC overall, a finding that remains consistent even as mPAP increases.^20^ The association of male sex with reduced RV function may explain why we observed that Group 3 PH patients have more RV impairment than PAH patients despite having less severe pulmonary vascular disease^8^ because our Group 3 PH cohort has only a slight female predominance (52%) (versus an ≈4:1 female predominance in PAH).^30^, ^31^, ^32^, ^33^

There is strong preclinical and clinical data that demonstrate that sex hormones contribute to the sex differences in RV function. In particular, there is evidence that testosterone plays a maladaptive role in RV pressure overload, while estrogen preserves RV function in PAH models. First, in male pulmonary artery–banded mice, castration results in less pathological RV remodeling and fibrosis, which is prevented by testosterone replacement.^34^ The improvements in RV structure with castration are associated with enhanced survival in male pulmonary artery–banded mice.^34^ In Sugen‐5416 (SU‐5416) hypoxia rats, 17β‐estradiol supplementation blunts RV remodeling and augments exercise capacity in both male and female rats, likely mediated through anti‐inflammatory properties and inhibition of RV cardiomyocyte apoptosis.^35^ Furthermore, in SU‐5416 hypoxia mice, treatment of ovariectomized female mice with 17β‐estradiol improves RVEF and RV‐pulmonary artery coupling.^36^ Moreover, in SU‐5416 hypoxia ovariectomized female rats, 17β‐estradiol replacement preserves exercise capacity and increases cardiac output.^37^ However, this study did demonstrate that 17β‐estradiol blunts pulmonary vascular remodeling, so the improvement in RV function may be partially because of a reduction in pulmonary vascular disease severity.^37^ At the molecular level, 17β‐estradiol administration to SU‐5416 hypoxia female rats prevents the reduction in mitochondrial mass and oxidative capacity, which is proposed to be mediated via increased expression of peroxisome proliferator‐activated receptor‐γ coactivator (PGC)‐1α.^38^ In the MESA cohort, estrogen replacement therapy results in higher RVEF when compared with women without estrogen replacement.^39^ Whether the male disadvantage reflects the double hit of too much androgen and insufficient estrogen remains to be definitively established, although this is suggested by the available literature. In summary, sex hormones, though not measured in our cohort, are strongly linked to RV function and adaption, and future studies may elucidate how manipulation of sex hormones may combat RV dysfunction in Group 3 PH and beyond.

As for why there are disproportionate decreases in RV function in both sexes in Group 3 PH, we evaluated the possibility that this might relate to the chronic hypoxia that characterizes this syndrome. Chronic hypoxemia has been shown to depress ventricular function.^40^, ^41^ Hence, it is possible that the disproportionate RV dysfunction in Group 3 PH is caused by an afterload‐independent effect of chronic hypoxemia on the RV. To test this, we assessed the association between RVFAC and supplemental oxygen requirement, as well as systemic oxygen saturation at rest. In our analysis, both supplemental oxygen requirement (HR: −2.3, 95% CI: −6.77–2.21, *P*=0.316) and systemic oxygen saturation at rest (HR: 0.18, 95% CI: −0.95–1.30, *P*=0.757) were not associated with RVFAC. This was true when we analyzed the association between supplemental oxygen requirement (HR: 0.06, 95% CI: −0.16–0.28, *P*=0.608) and systemic oxygen saturation (HR: −0.03, 95% CI: −0.08–0.03, *P*=0.356) with RVFAC as a binary variable. Thus, hypoxemia does not appear to mediate RV dysfunction in Group 3 PH; however, we did not have data about nocturnal hypoxemia so we cannot completely rule out a relationship between hypoxemia and RV function.

Our findings that RV dysfunction increases the risk of heart failure hospitalization and death in Group 3 PH is congruent with the findings that reduced RVEF on cardiac MRI is a univariate predictor of mortality in a Scottish cohort of severe Group 3 PH patients.^42^ These results suggest that therapies that improve RV function may be beneficial. If this were proved to be the case, it would be an important advance because we currently have very little to offer this patient population because PAH‐specific therapy does not consistently reduce symptom burden or enhance exercise capacity.^7^ Currently, the only treatment for Group 3 PH is lung transplantation, and with the limited availability of donor lungs and rising prevalence of Group 3 PH,^2^ it is vital that novel treatments be developed. Our data suggest that RV‐directed therapies may combat heart failure hospitalizations and death in Group 3 PH, and future studies investigating such therapies should be conducted.

### Limitations

Our study has several limitations. This is a single‐center longitudinal observational cohort study. Nearly half of the patients were entered into our registry retrospectively. However, this is one of the largest studies to comprehensively describe RV function in patients with Group 3 PH. In addition, we collected the baseline variables at the time of the original referral to our practice instead of the variables at the time of enrollment in the registry. This approach reduces survival bias. Furthermore, there are no significant differences in clinical characteristics and outcomes between the incident and the prevalent cohort in our registry (data not presented). We did not have raw echocardiogram data on all patients to quantify RV function and some patients did not have quantifiable images. RVFAC has more variability than TAPSE,^43^ but we showed acceptable reproducibility with intraobserver variability correlation coefficient of 0.95 and a mean difference of −0.6±3.3%. We could only analyze RV hemodynamic tracings from patients from 2014 onward; thus, not all patients could have the RV contractility and Tau analysis performed, leading to missing data. As we performed multiple comparison in our analysis, considering a *P*<0.05 as significant may have resulted in some false‐positive results. To account for this, we have presented all *P* values in 3 decimals. Finally, most of the Group 3 patients in our cohort had severe PH and thus, our data reflect referral bias to an academic PH program and is not representative of a population‐based Group 3 PH cohort.

## Sources of Funding

Prins is funded by NIH K08 HL140100, Archer is supported by Canada Foundation for Innovation (229252 and 33012), NIH RO1 HL113003, a Tier 1 Canada Research Chair in Mitochondrial Dynamics and Translational Medicine (950‐229252), the Queens Cardiopulmonary Unit (QCPU), and a grant from the William J Henderson Foundation. Thenappan is funded by American Heart Association Scientist Development Grant 15SDG25560048.

## Disclosures

Dr Thenappan received modest consultation fees from Actelion and Gilead. The remaining authors have no disclosures to report.
